# Membrane Lipids in Ultra-High-Risk Patients: Potential Predictive Biomarkers of Conversion to Psychosis

**DOI:** 10.3390/nu15092215

**Published:** 2023-05-07

**Authors:** Ariel Frajerman, Boris Chaumette, Dominique Farabos, Gaétan Despres, Christelle Simonard, Antonin Lamazière, Marie-Odile Krebs, Oussama Kebir

**Affiliations:** 1 Institute of Psychiatry and Neuroscience of Paris (IPNP), Université de Paris, INSERM U1266, F-75014 Paris, France; 2 GHU Paris Psychiatrie et Neurosciences, F-75674 Paris, France; 3 Department of Psychiatry, McGill University, Montréal, QC H3A 0G4, Canada; 4 INSERM UMR S 938, Département METOMICS, Centre de Recherche Saint-Antoine, Sorbonne Université, AP–HP, F-75012 Paris, France

**Keywords:** membrane lipids, fatty acids, omega-3, psychosis, ultra-high risk

## Abstract

Alterations in membrane lipids are reported in schizophrenia. However, no conclusion can be drawn regarding the extended and predictive value of these alterations in persons at ultra-high risk of psychosis (UHR). Recent studies suggested that sterols’ impact on psychiatric disorders was underestimated. Here, we simultaneously explored sterols, fatty acids (FA), and phospholipids (PL) in UHR persons for the first time. We analysed erythrocyte membrane lipids in 61 UHR persons, including 29 who later converted to psychosis (UHR-C) and 32 who did not (UHC-NC). We used gas chromatography for **FA** and liquid chromatography tandem with mass spectrometry for sterols and phospholipids. Among UHR individuals, elevated baseline membrane linoleic acid level was associated with conversion to psychosis (26.1% vs. 60.5%, *p* = 0.02). Combining sterols, FA, and PL membrane composition improved the prediction of psychosis onset (AUC = 0.73). This is the first report showing that membrane sterol participates, with other membrane lipids, in modulating the risk of psychosis. It suggests that membrane lipids could be used as biomarkers for personalised medicine in UHR patients.

## 1. Introduction

**Figure 1:**
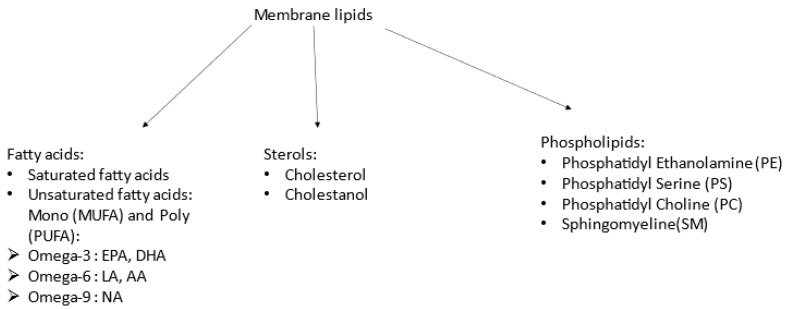
Schematic representation of membrane lipids’ composition. EPA: Eicosapentaenoic acid; DHA: docosahexaenoic acid; LA: linoleic acid; AA: arachidonic acid; NA: nervonic acid.

The plasma membrane is a complex system regulating not only intracellular and extracellular membrane exchanges but also the synaptic release of many neurotransmitters, receptor activity, and many other biological processes, such as inflammation and oxidative stress [1]. The plasma membrane is made up of sterols (mainly cholesterol but also cholestanol) and phospholipids (PLs, phosphatidylethanolamine (PE), phosphatidylcholine (PC), phosphatidylserine (PS), and sphingomyelin (SM)), as well as fatty acids (FAs), including polyunsaturated (PUFAs: omega-3, omega-6, and omega-9) and saturated FAs (Figure 1). Proportions of each vary regarding cell type [2]. Neurotransmitter receptors are embedded in neurons’ membranes and interact with their lipid environment, which modulates their activity, especially receptors for glutamate (NMDA (N-methyl D-aspartic acid), AMPA (α-amino-3-hydroxy-5-methyl-4-isoxazole propionic acid)), GABA (γ-Aminobutyric acid), and 5HT (serotonin) [3]. In the presynaptic membrane, PUFA composition could impact vesicle formation [4]. Therefore, membrane composition influences synaptic transmission [5]. Knowledge of the importance of cholesterol for membrane metabolism is recent; it has been shown to be involved in maintaining the asymmetry of membrane phospholipids between the inner and outer leaflets [6]. This asymmetry is important for the normal function of erythrocytes [7]. In the same year, another team found a linear correlation between Na+/K+-ATPase activity and membrane cholesterol level (no link with serum cholesterol). The activity of this enzyme is inversely correlated with the level of TBARS (thiobarbituric acid reactive substances), a marker of oxidative stress [8].

Schizophrenia is a common psychiatric illness [9] that usually begins between 15 and 25 years of age. It has multifactorial determinants, including genetic predisposition, as well as early and late environmental factors, including childhood adversities, stress, and substance consumption. Cannabis is the most documented risk factor triggering psychosis and could disrupt physiological brain maturation processes driven by the endocannabinoid system [10]. Attenuated or transient symptoms predate full-blown psychosis [11], and criteria were defined to distinguish persons at ultra-high risk (UHR) [12], either experiencing attenuated psychotic symptoms (APS), brief, limited intermittent psychotic episodes (BLIP), and/or trait vulnerability plus a marked decline in functioning [13]. According to a recent meta-analysis, 25% of UHR patients developed psychosis within 3 years [14]. Of crucial interest, identifying individuals at risk of psychosis could open the field to specific preventive interventions.

For the pathophysiology of schizophrenia, the current hypothesis is that the pathology appears, on a predisposing genetic ground, according to the exposure to certain environmental factors; whether they appear during pregnancy, early childhood, or adolescence. The triggering of the first psychotic episode is, therefore, the result of a gene–environment interaction [15]. Data from the literature have highlighted numerous biological abnormalities: in the dopaminergic [16], glutamatergic [17], and endocannabinoid [18] signalling pathways, oxydative stress [19], inflammation [20], membrane lipids [21], etc.

Abnormalities in membrane lipids have long been studied and led Professor Horrobin to formulate the membrane hypothesis of schizophrenia. According to him, schizophrenia is due to an abnormality in the membrane biochemistry, leading to an abnormality in the membrane structure, expressed in many membranes in the body, including those of neurons. An abnormality in membrane biochemistry is an abnormality in synthesising phospholipids that are incorporated into membranes and continuously remodelled. It is related to the FA metabolism in the membranes [22]. However, these membrane lipid abnormalities are not found in all schizophrenic patients, but a bimodal distribution of lipid concentrations in patients suffering from schizophrenia has been found by several independent studies [23,24,25]. Two subgroups can be distinguished: one with a deficiency of PUFAs or an abnormality in membrane phospholipids, and the other with a lipid profile similar to that of healthy subjects [23,24]. For instance, in a previous study of patients with chronic schizophrenia, we identified two subgroups; those with membrane sphingomyelin deficiency had more cognitive impairment and greater severity of the disease [25].

All the studies above focused on fatty acids and phospholipids, but sterols are very important for membrane function. A decrease has been reported in cholesterol from skin fibroblasts’ membranes of untreated first-episode psychosis (FEP) patients compared to control subjects [26]. In UHR patients, no study has so far investigated the membrane composition by simultaneously measuring sterols, phospholipids, and FA.

Our hypothesis is that there is an abnormality in membrane lipids in some UHR patients. This abnormality may be a biomarker of vulnerability to psychosis and present before the psychotic transition. 

## 2. Methods and Materials

### 2.1. Clinical Population and Assessments

This study is part of the ICAAR (“Influence du Cannabis sur l’émergence de symptômes psychopathologiques des Adolescents et jeunes Adultes présentant un état mental à Risque” (Influence of Cannabis on the Emergence of Psychopathological Symptoms in Adolescents and Young Adults with Mental Risk Conditions)) study (Promotion Groupe Hospitalier Universitaire Paris Psychiatrie & Neurosciences (GHU), PI Pr MO Krebs), detailed elsewhere [27]. In brief, young adults (both male and female, 16–30 y.o.) who were referred to the specialised outpatient clinic Adolescent and Young Adults Assessment Centre (‘Centre d’évaluation du Jeune adulte et de l’Adolescent’, C’JAAD) between 2009 and 2014 were included if they did not have a previous diagnosis of psychosis, current treatment with an antipsychotic (>12 weeks), severe substance misusage during the last year and/or for more than five years, severe or evolutive somatic and neurological disorders, head injury, and/or insufficient intellectual or linguistic skills. After signing their informed consent, help-seekers (*n* = 384) underwent a comprehensive sociodemographic, clinical, and cognitive assessment at baseline (M0), including the Comprehensive Assessment for At-Risk Mental State (CAARMS) [28]. Those characterised as UHR (CAARMS criteria [29]) were included in a one-year follow-up. The conversion was defined when they reached the “psychosis threshold” of the CAARMS. All study procedures were conformed to the Ethic Committee (CPP Ile de France 3). UHR individuals who convert to psychosis are called converters (UHR-C), and the others non-converters (UHR-NC).

### 2.2. Blood Samples

Red blood cell (RBC) samples were stored at −80 °C in the Biological Resource Center NSPN, GHU Paris Psychiatrie & Neurosciences. After quality controls to ensure the absence of lipid peroxidation, we collected available samples at inclusion (M0) and 6 months (M6) or at the final time (M12/MF). Among subjects initially enrolled, blood samples were still available for a subsample of patients: at inclusion: N = 61 (32 converters (C) and 29 non-converters (NC)) and at the second time: N = 54 (23 C and 31 NC). To improve the statistical comparisons, this subsample was greater in converters (almost 50%), and thus was not representative of the initial population.

### 2.3. Lipid Analysis

We used the same method as previously described [25]. In brief, total lipids were extracted from the RBC cell membranes using Folch’s method [30]. Samples were split into 3 parts. Fatty acid levels were measured with gas chromatography [31]. Phospholipids [32,33] and sterols (cholesterol and cholestanol) [34] were measured with liquid chromatography tandem mass spectrometry (LC-MS/MS). 

Lipids analysis was conducted at the Mass Spectroscopy Department at the Saint-Antoine Hospital, Paris, France. Internal standards for the determination of phospholipids, sterols, and sphingolipids are from Avanti Polar Lipids (Alabaster, AL, USA). Ammonium acetate and trimethylsilyl diazomethane came from Sigma-Aldrich (Saint Quentin Fallavier, France). Methanol, chloroform, n-hexane, heptane, and isopropanol of mass spectrometry quality were obtained from VWR (Fontenay Sous-Bois, France). Total lipids were extracted from the RBC cell membranes based on the methods of Folch et al. Samples were divided into 3 parts.

- Fatty acids: After extraction, they were then trans-methylated in acid conditions and separated by gas chromatography–mass spectrometry using a Thermo GC/MS/FID TRACE DSQ2 device.
- Phospholipids: Lipid extracts were suspended in 200 µL cyclohexane/isopropanol/water/ammonium acetate, 500 mM (58/40/0/2) volume (Solvent A). For phospholipid analysis, a total of 10 µL was injected onto a 3.0 mm × 250 mm length PVA-SIL column (YMC Europe GmbH, D-46514, Schermbeck, Germany), at a flow rate of 150 µL/min, with a total run time of 70 min. A 2 mm frit cap and a short reverse-phase guard cartridge (in-line guard C18-silica, 3 µm, 4 × 20 mm^2^, CIL-Cluzeau, 92419, Courbevoie, France) were used to prevent the capillary clogging. A passage through the guard cartridge was used to decrease ion suppression. The mobile phase gradient used consisted of solvent A and solvent B (cyclohexane/isopropanol/water/ammonium acetate 500 mM (50/40/8/2). In each measurement, gradient elution was applied to separate each lipid class. Both the application of HPLC solvent gradient and mass spectrometer scan functions were controlled by the Analyst Software (AB Sciex) data system. The samples were analysed using an electrospray ionisation tandem mass spectrometry (ESI/MS/MS, 6500 ABSciex, TQ, Applied Biosystems-Sciex, Concord, ON, Canada) either with scan mode or multiple-reaction monitoring (MRM). The specific detection of lipid classes was based on the mass-to-charge ratio (*m*/*z*) value of their precursor ion scanning, which was related to their head group fragments. The scans were conducted in negative-ion mode. Based on the precursor ion scanning value, the PL was identified at 184 (*m*/*z*) for PC and SM, 185 (*m*/*z*) for PS, and neutral loss scanning 141 (*m*/*z*) for PE. A comprehensive description of the methodology can be found in Lamazière et al. [32].
- Sterols: Sterols were extracted with a solvent mixture containing chloroform/methanol 2/1 (*v*/*v*) spiked with internal standards. Lipids were partitioned in chloroform after the addition of saline and evaporation under nitrogen, and saponified by methanol potassium hydroxide. The fatty acids released were then methylated with BF3-methanol to prevent them from interfering with the chromatography of sterols. Sterols were further re-extracted in hexane and silylated, with evaporation under nitrogen; then, we added 150 µL cyclohexane 10% BSTFA and the resultant derivatives were separated by gas chromatography (GC) (Hewlett–Packard 6890 series) in a medium-polarity capillary column RTX-65, (Restesk, Evry, France). The mass spectrometer (Agilent 5975 inert XL) in series with the GC was set up for the detection of positive ions, which were produced in the electron impact mode at 70 eV. Sterols were identified by the fragmentogram in the scanning mode and quantified by selective monitoring of the specific ions after normalisation with the internal standards and calibration with weighed standards. For more detailed descriptions, see Chevy et al. [35].

### 2.4. Statistical Analysis

Comparisons of means were conducted with a Wilcoxon test method (non-parametric test). Adjustment for multiple tests was performed with the Benjamin Hochberg method. We used Spearman’s correlation (non-parametric test) for correlations between lipids. These analyses were conducted on the R software version 4.0, using packages *readxl*, *corrplot*, *Cairo*, *RColorBrewer*, and *stats*.

We performed principal component analyses (PCA) with logistic regression on the PCA factors (unsupervised analysis) with adjustment for age and sex and ROC curves to assess the quality of prediction. As in a previous exploratory study, we tested several combinations of lipid species [36,37]. We used a clustering analysis with the variables highlighted by the ROC curves. The clustering was conducted using the two-step method. These analyses were performed on SPSS© version 20 software.

We used the website https://www.metaboanalyst.ca/MetaboAnalyst/ (accessed on 29 April 2023) to create the heatmaps, excluding missing data and showing only group averages.

## 3. Results

In our sample, there were no differences in sociodemographic or clinical characteristics, or plasmatic lipid values between converters and non-converters at the time of inclusion (Table 1). Membrane lipids did not correlate with their body mass index or plasmatic cholesterol levels. FA levels were correlated with triglyceride levels (Table 2).

### 3.1. Comparison at Baseline between Converters and Non-Converters

**Figure 2:**
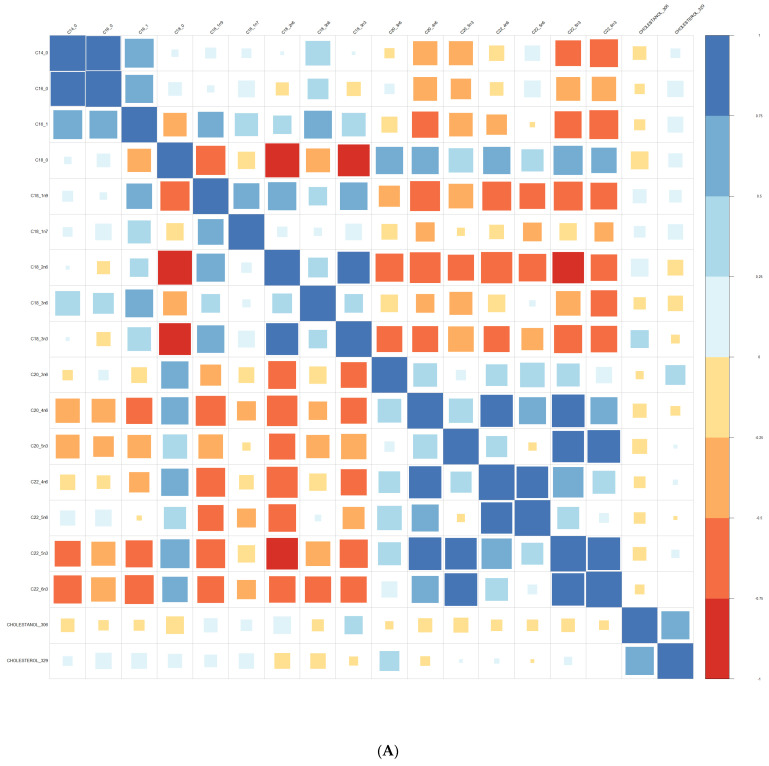

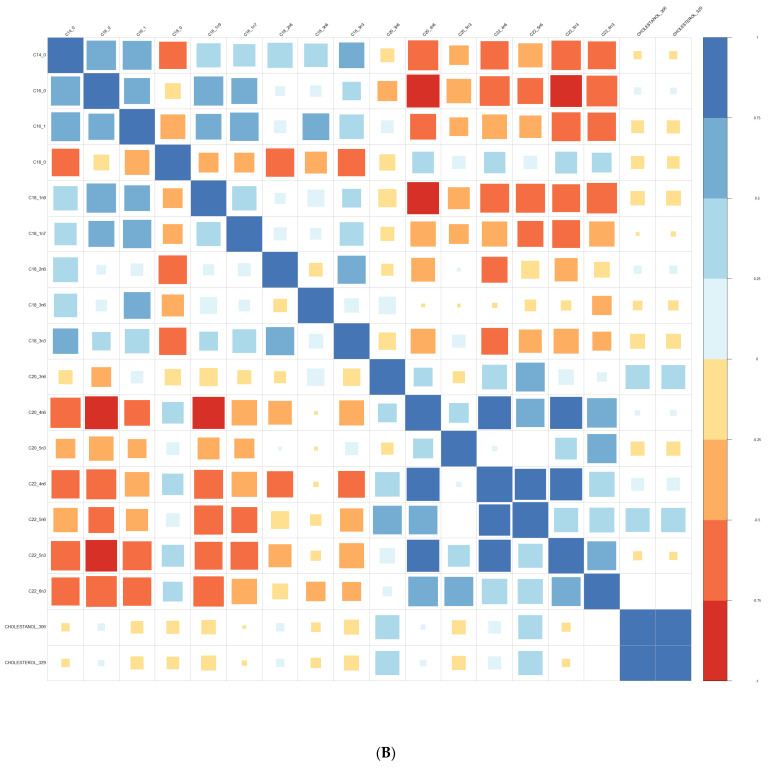
Correlation matrix of fatty acids and sterols at baseline in converters (**A**) and non-converters (**B**). White: no significant correlation (*p* > 0.05). Blue: positive correlations. Yellow/orange/red: negative correlation. The intensity of the colour and the size of the squares are proportional to the correlation coefficients. There was no correction for multiple comparisons.

**Figure 3:**
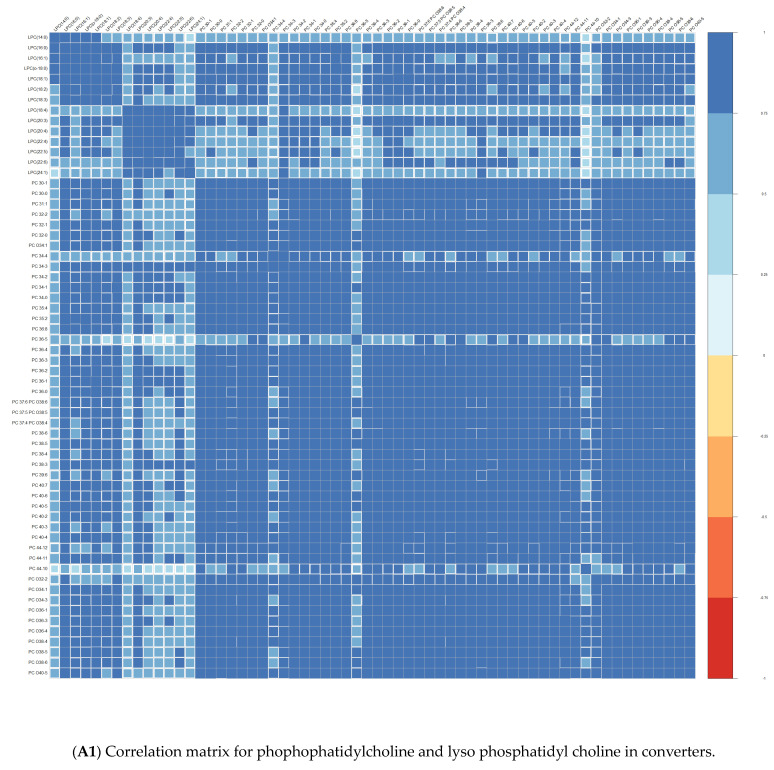

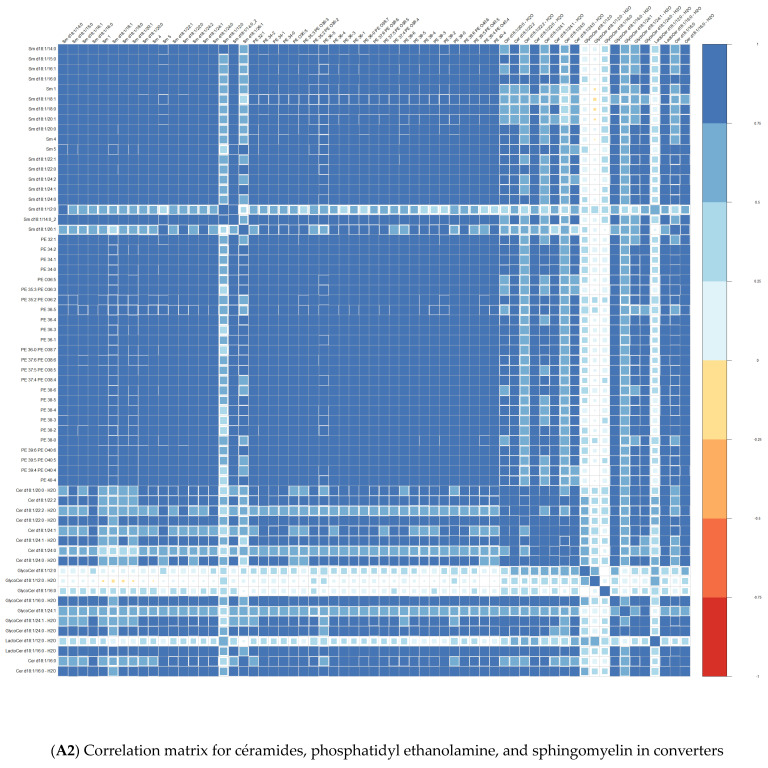

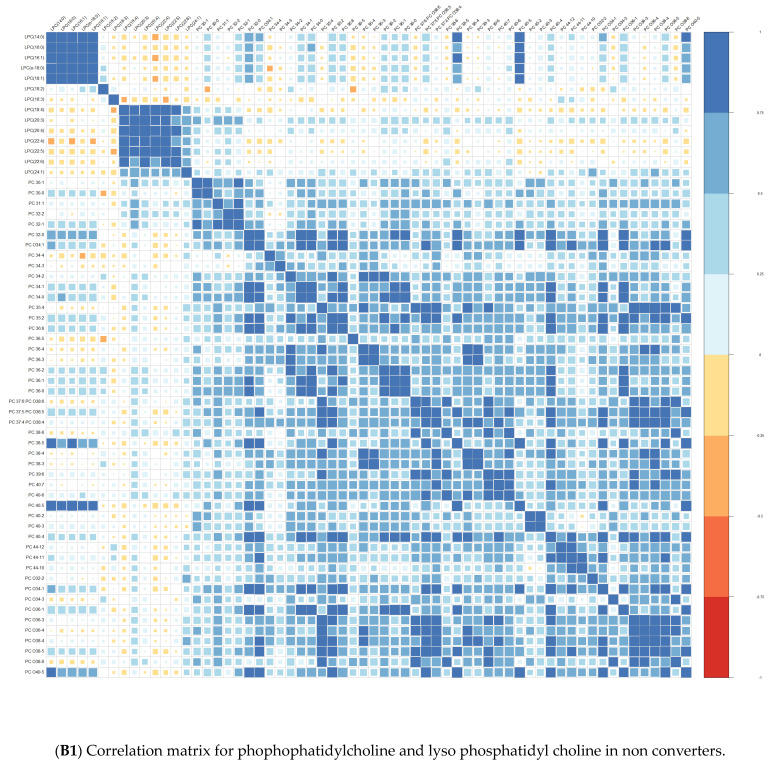

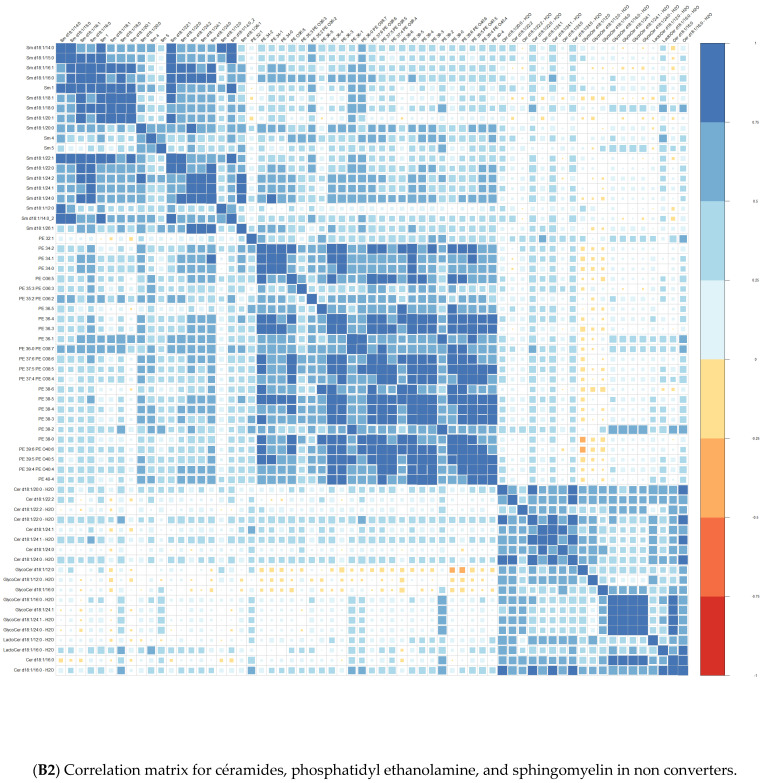
Correlation matrix of phospholipids at baseline in converters (**A1**,**A2**) and non-converters (**B1**,**B2**). White: no significant correlation. Blue: positive correlations. Yellow/orange/red: negative correlation. The intensity of the colour and the size of the squares are proportional to the correlation coefficients. There was no correction for multiple comparisons. Cer: Céramides; Glyco Cer: Glyco Céramides; Lacto Cer: Lacto Céramides; LPC: Lyso Phosphatidyl Choline; PC: Phosphatidyl Choline; PE: Phosphatidyl Ethanolamine; SM: Sphingomyelin.

The lipid patterns were different between converters and non-converters before psychotic conversion (Figure 2 and Figure 3). At baseline, the mean lipid percentages were nominally significantly different between future converters and non-converters regarding 14 types (LactoCer d18:1/16:0 H_2_O, PE 36-0 PE O38:7, PE 36-1, PE 34-0, GlycoCer d18:1/16:0—H_2_O, PS 32:0, PE 34-1, PE 34-2, PE 36-4, Cer d18:1/22:2—H2O, PE 38-2, PE 38-6, C18_2n6, PE 32:1). The differences were larger in PE and linoleic acid (C18:2n6). Heatmaps are in Supplementary Figure S1 (fatty acids) and Figure S2 (phospholipids). There was no difference after adjustment for multiple testing.

The comparison of the mean lipid percentages between converters and non-converters after the follow-up showed differences for five species, mainly omega-6 (C20_3n6, PC 44-12, C18_3n6, C22_5n6, C16_0). There was no difference after adjustment for multiple testing. 

### 3.2. Prediction of Conversion to Psychosis

**Figure 4:**
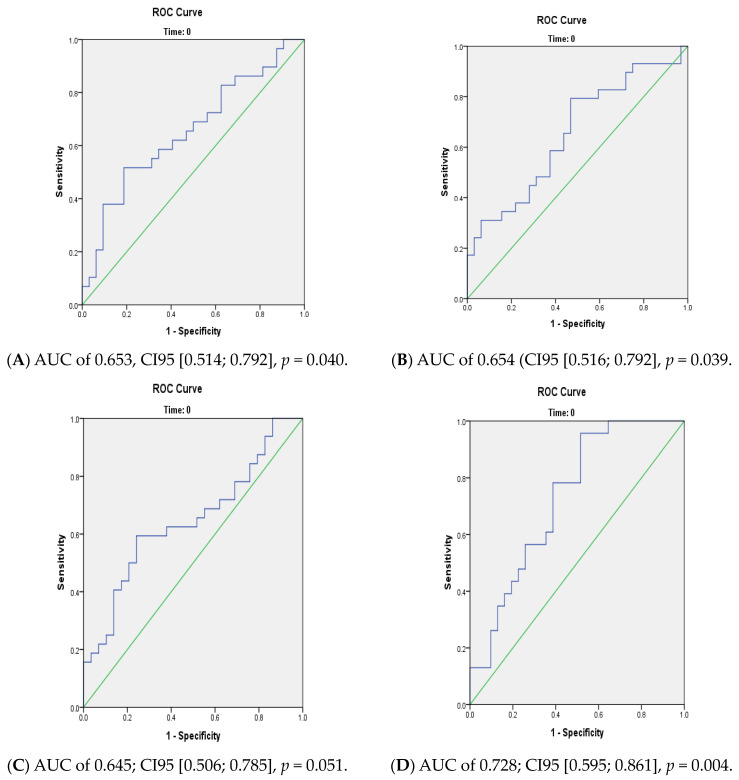
ROC curves obtained from all fatty acids (**A**) and linoleic acid alone (**B**), all phospholipids (**C**), and the combination of linoleic acid + phosphatidylcholine (PC) species + cholestanol/cholesterol ratio (**D**).

We conducted a logistic regression after a reduction in dimensions using a principal component analysis to predict the psychotic conversion using measures at inclusions. Using the linoleic acid (LA) level alone produced a similar result (AUC of 0.654 (CI95 [0.516; 0.792], Figure 4B) to using all FA levels (AUC of 0.653, CI95 [0.514; 0.792], Figure 4A) or using all the phospholipid levels (PL, AUC of 0.645; CI95 [0.506; 0.785], Figure 4C). The best prediction was obtained by combining LA, phosphatidylcholine species, and the cholestanol/cholesterol ratio (AUC of 0.728 (CI95 [0.595; 0.861]), Figure 4D). 

**Figure 5:**
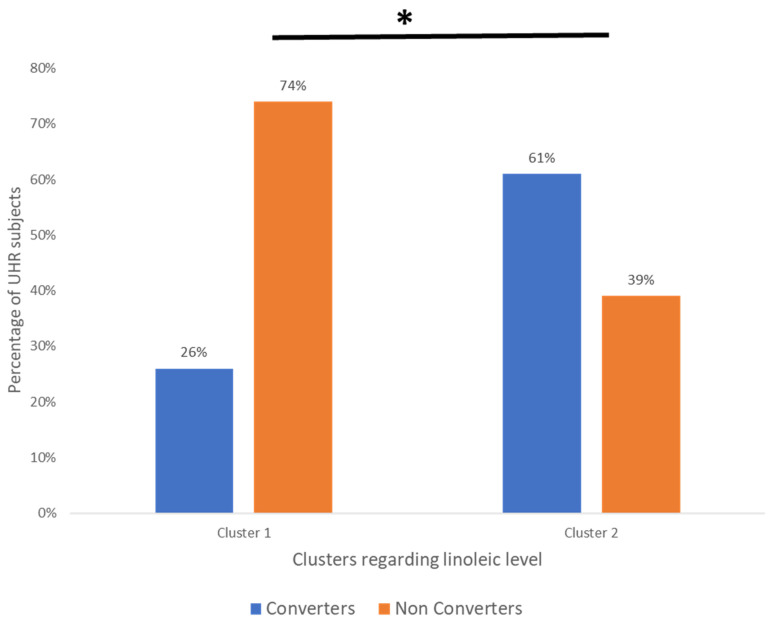
Clustering regarding the linoleic acid (LA) level at baseline using the 2-step clustering method. * *p* < 0.05. Cluster 1: low LA level. Cluster 2: high LA level.

**Figure 6:**
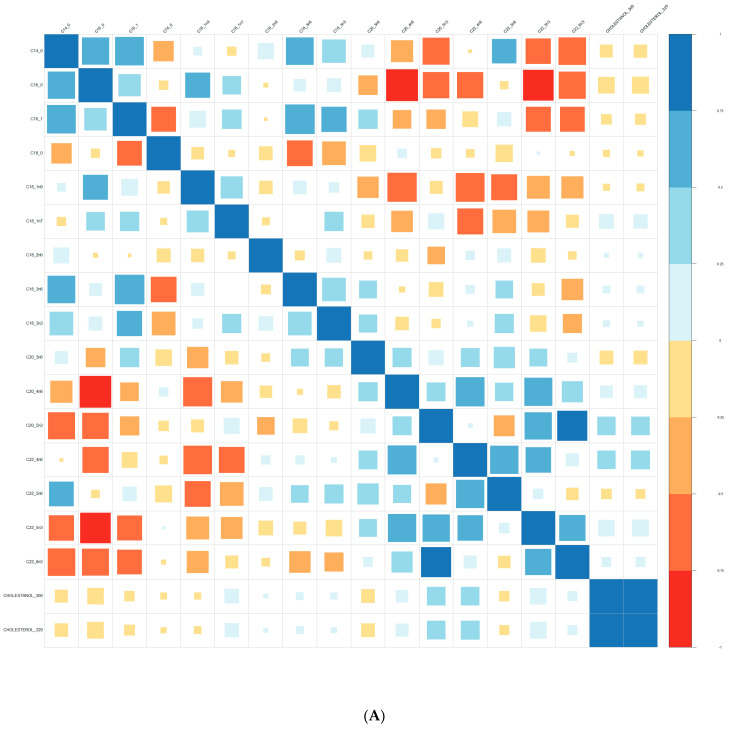

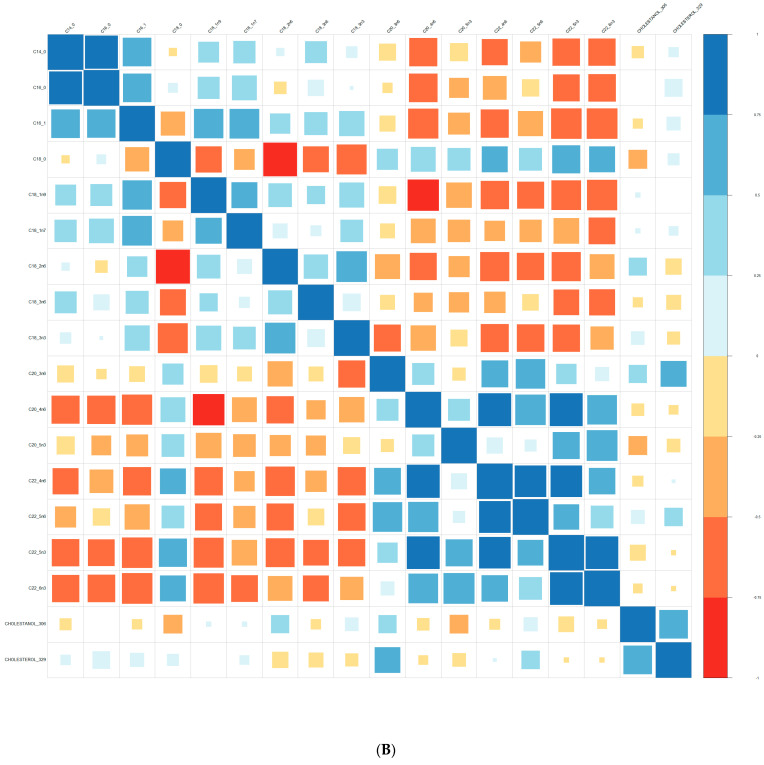
Correlation matrix of fatty acids and sterols at baseline for subject cluster with low level of linoleic acid (**A**) and cluster with high level of linoleic acid (**B**). White: no significant correlation (*p* > 0.05). Blue: positive correlations. Yellow/orange/red: negative correlation. The intensity of the colour and the size of the squares are proportional to the correlation coefficients. There was no correction for multiple comparisons.

Clustering was carried out using the level of linoleic acid (LA, omega-6), distinguishing two subgroups of patients (clustering): those with a low LA level have a risk for psychosis lower than that of subjects with a high LA level (26.1% vs. 60.5%, *p* = 0.02) (Figure 5 and Table 3). The two subgroups exhibit other significantly different membrane PUFA levels (higher levels of arachidonic acid (C20:4n6), docosatetraenoic acid (C22:4n6), and docosapentaenoic acid (C22:5n-3) and lower levels of α-linolenic acid (C18:3n3) in the subgroup with the lowest risk of psychosis) (Table 4). There are also differences in fatty acid patterns (Figure 6). Heatmap is in Supplementary Figure S3.

We also conducted clustering on the cholestanol/cholesterol ratio, but it was not significant (*p* = 0.71).

### 3.3. Lipid Composition over Time 

In the whole sample, longitudinal changes in mean lipid percentages were found between the inclusion time and the final time for five species: gamma-linolenic acid (C18_3n6), PS 36:0, LPC (18:3), C16_0, PS 32:0 (Table 5). After adjustment, significant differences only persisted for gamma-linolenic acid and PS36:0. 

In converters, the longitudinal changes in lipid compositions were seen in five species: gamma-linolenic acid, PS 36:0, C16_0, PS 32:0, and Cer d18:1/24:1, whereas in non-converters, the longitudinal changes in lipid composition were found for three species: PS 36:0, C16_0, and C22_5n6. These differences did not persist after adjustment for multiple testing. Heatmaps for change over time are in Supplementary Figure S4 for all subjects, Supplementary Figure S5 for converters, and Supplementary Figure S6 for non-converters.

The longitudinal changes in lipid composition were different between converters and non-converters for seven species: C22_5n6, PC O34:1, PC 38-3, LPC(18:3), Cer d18:1/16:0—H2O, Cer d18:1/22:2—H2O, and LactoCer d18:1/12:0 (Table 6). These differences did not persist after adjustment for multiple testing. 

**Figure 7:**
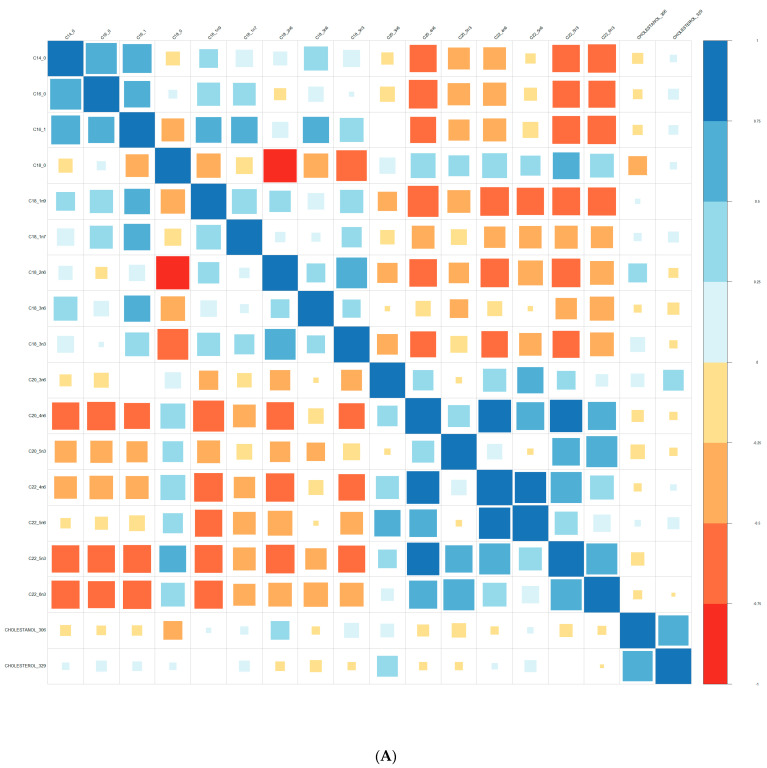

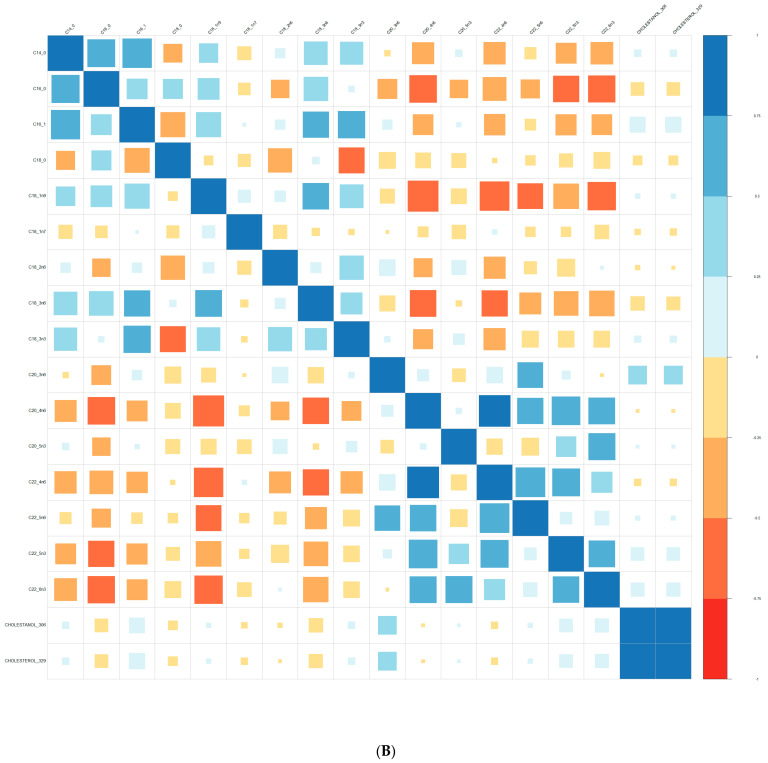
Correlation matrix of fatty acids and sterols for all subjects (converters + non-converters) at baseline (**A**) and final time (**B**). White: no significant correlation (*p* > 0.05). Blue: positive correlations. Yellow/orange/red: negative correlation. The intensity of the colour and the size of the squares are proportional to the correlation coefficients. There was no correction for multiple comparisons.

**Figure 8:**
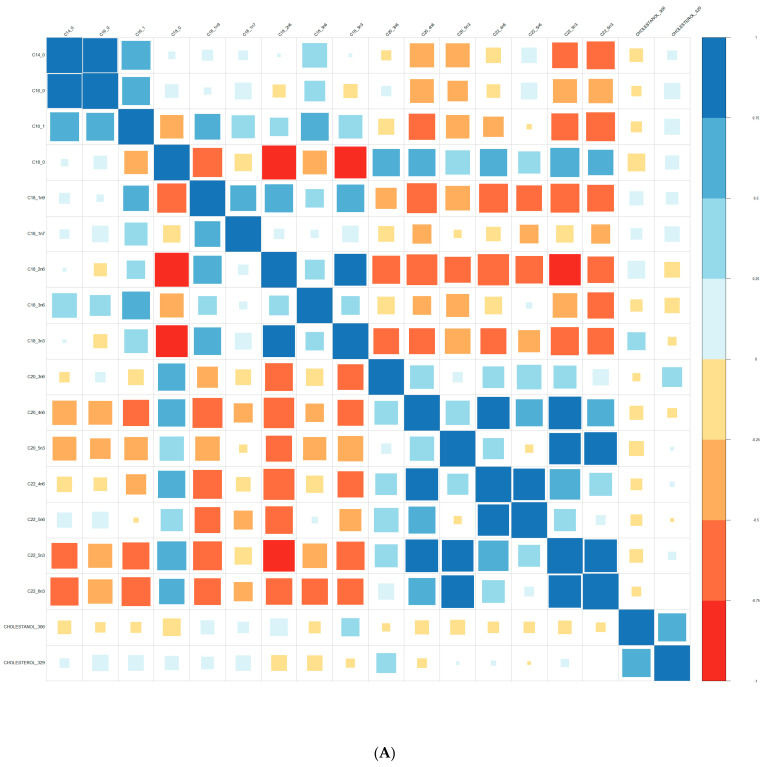

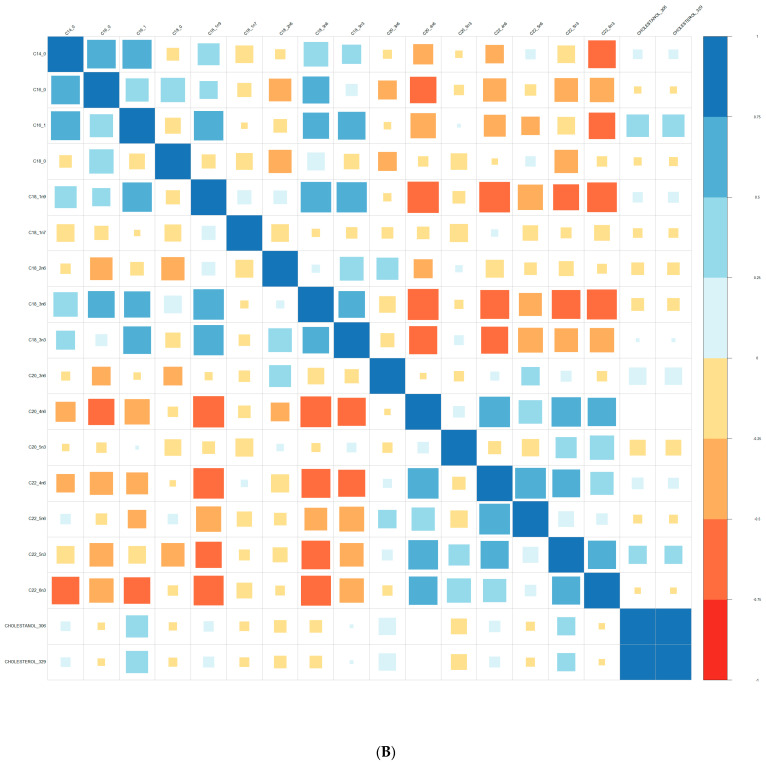
Correlation matrix of fatty acids and sterols in converters at baseline (**A**) and final time (**B**). White: no significant correlation (*p* > 0.05). Blue: positive correlations. Yellow/orange/red: negative correlation. The intensity of the colour and the size of the squares are proportional to the correlation coefficients. There was no correction for multiple comparisons.

**Figure 9:**
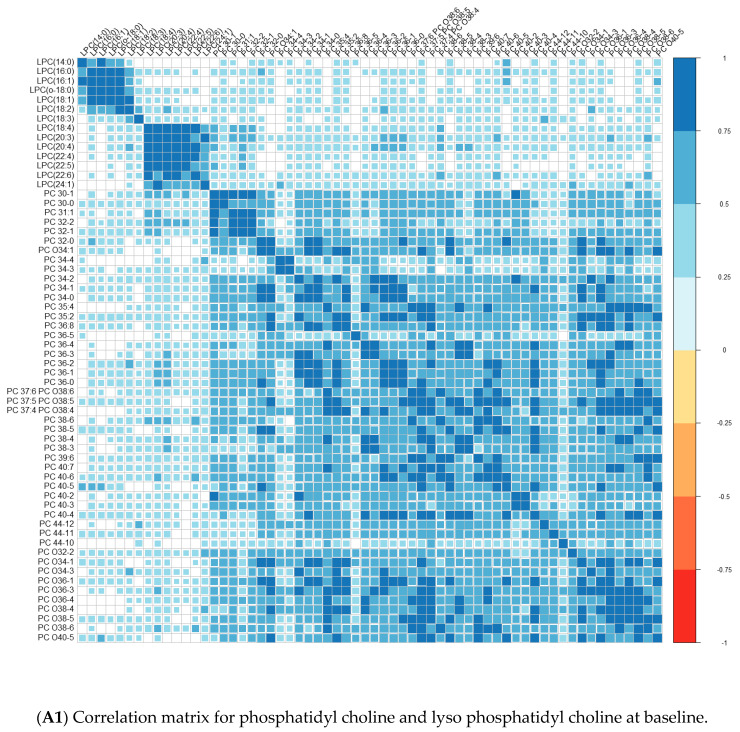

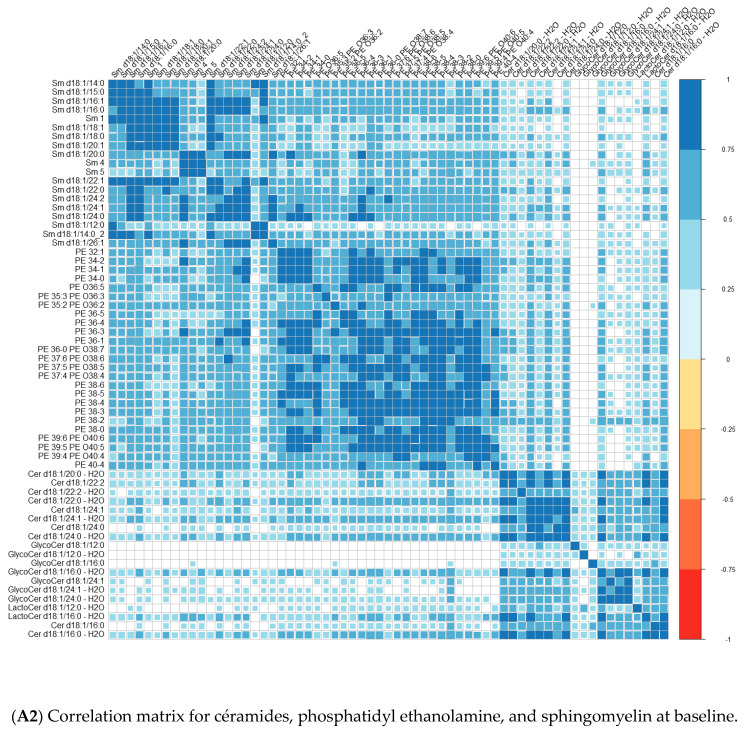

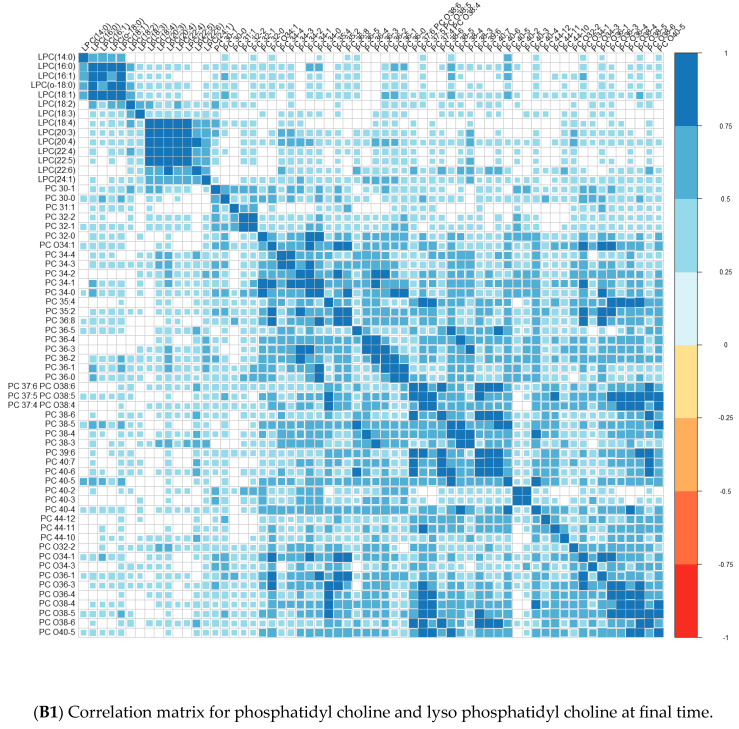

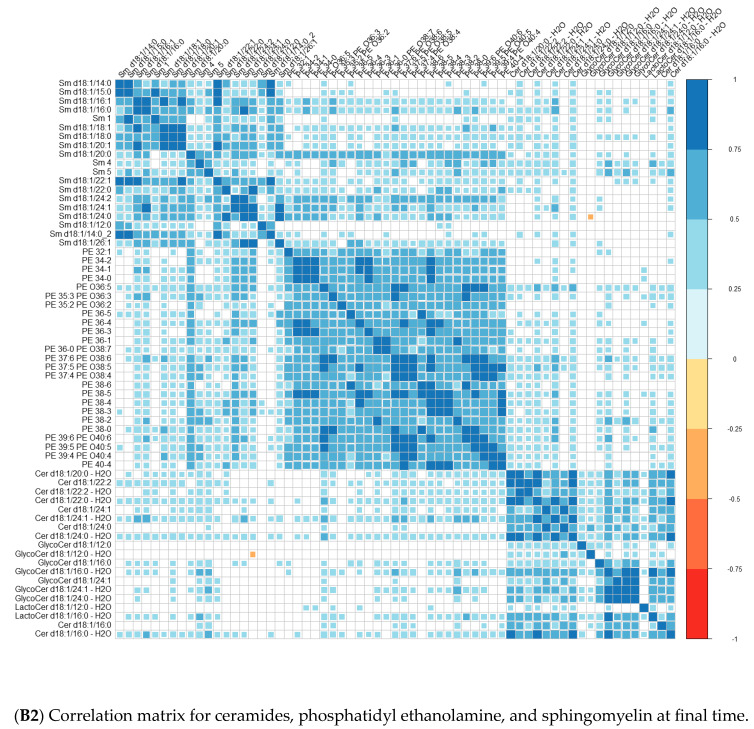
Correlation matrix of phospholipids at baseline (**A1**,**A2**) and final time (**B1**,**B2**). White: no significant correlation. Blue: positive correlations. Yellow/orange/red: negative correlation. The intensity of the colour and the size of the squares are proportional to the correlation coefficients. There was no correction for multiple comparisons. Cer: Céramides; Glyco Cer: Glyco Céramides; Lacto Cer: Lacto Céramides; LPC: Lyso Phosphatidyl Choline; PC: Phosphatidyl Choline; PE: Phosphatidyl Ethanolamine; SM: Sphingomyelin.

There are differences between the fatty acid correlation patterns (matrix correlations using the Spearman method) at inclusion and the final time (Figure 7), particularly in the converters (Figure 8) and also for phospholipids (Figure 9).

## 4. Discussion

For the first time, we simultaneously tested the value of many membrane lipids, including sterols, fatty acids, and phospholipids, as markers of risk of psychosis.

We found differences between converters and non-converters in lipid patterns before conversion to psychosis for linoleic acid (precursor of the omega-6 family). We also found differences between converters and non-converters after the psychosis onset for three other FA of the omega-6 family. Hence, the evolution over time was different between converters and non-converters, particularly for omega-6, which may suggest differences in metabolism between the two groups. In our cohort, using linoleic acid at baseline allowed us to distinguish two subgroups with a significantly different percentage of converters, those being enriched in the cluster with a higher linoleic acid level (26.1% vs. 60.5%). The value of membrane lipids as a biomarker to predict conversion to psychosis remains relatively limited. However, using all membrane lipids (FA, PL, and sterols) allows for a better prediction (AUC: 0.73) than considering only a single type of membrane lipid.

### 4.1. Membrane Lipids as Biomarkers of Conversion to Psychosis in UHR

Previous reports already support the value of membrane lipids [21] to discriminate between converters and non-converters. In the Vienna cohort exploring FA, the nervonic acid level (NA, omega-9) was significantly lower in converters than in non-converters. There was no difference for arachidonic acid (AA, omega-6) and docosahexaenoic acid (DHA, omega-3) [38]. In an independent multicenter cohort, NEURAPRO, the analysis explored FA and phospholipids using liquid chromatography and mass spectrometry (HPLC-MS). Compared to control subjects, eicosapentaenoic acid (EPA, omega-3), DHA, and AA levels decreased in UHR while NA levels increased. UHRs also showed an increase in sphingomyelin (SM) and a decrease in phosphatidylethanolamine (PE) levels [39]. However, the authors estimated the omega-3 levels and the omega-3 index but did not really measure them [40]. In the multicenter studies, patients came from many countries, whereas all controls only came from Australia; this may have biased FA levels, which are related to the geographical differences in the diets [41]. Our study extends previous reports on the predictive effect of FA, especially linolenic acid (C18:2n6). 

In comparison with these previous reports, our study included a larger number of converters (n = 29) than the Vienna (n = 11) and the NEURAPRO (n = 22) cohorts. Moreover, all subjects were recruited in France, providing more lifestyle and diet homogeneity. 

### 4.2. Linolenic Acid (LA: C18:2n6) and Schizophrenia

Linoleic acid (LA) is the precursor of omega-6 polyunsaturated fatty acids and the most highly consumed PUFA in the human diet [42]. A recent review concluded that excess LA in food might adversely affect the brain. Data on rodents suggested that LA may have an inflammatory effect on the brain. Data on newborns and children suggested a long-lasting impact of maternal LA on offspring’s cognitive skills and an increased risk of autistic traits [43].

In serum or plasma, LA may be associated with psychosis, as reported in a systematic review of metabolomics in patients with psychosis [44]. However, this review includes studies on serum, while our research is focused on membrane lipids. More recently, a two-sample Mendelian randomisation found that linoleic acid concentration was associated with an increased risk of schizophrenia (inverse variance weighted OR 1.06 [95% CI 1.01–1.12], *p* = 0.01). Estimates from the FADS single-SNP analyses also indicated that the short-chain omega-3 and omega-6 fatty acid concentrations were associated with an increased risk of schizophrenia (OR 1·08 [95% CI 1.02–1.15] for α-linolenic acid and 1.18 [1.04–1.36] for linoleic acid [45]. Furthermore, a study used a biomarker panel including C16 sphinganine, gamma-linolenic acid, linoleic acid, PC (16:0/18:1), PE (20:2/18:2), and sulfate to discriminate between first-episode schizophrenic patients before and after treatment by antipsychotics. They obtained an optimal classification performance with an AUC = 0.905 [95% CI 0.813–0.967] [37].

Regarding membrane, a meta-analysis including 18 cohorts (9 medicated, 4 antipsychotic-free, and 5 antipsychotic-naïve) reported that LA was decreased only in patients taking medication compared to control subjects [46]. Another meta-analysis found a differential effect of the medication on the LA levels depending on the type of antipsychotic drugs (typical versus atypical) [47]. 

In UHR patients from the Vienna cohort, the LA level was not associated with psychosis (*p* = 0.543) but with a mood disorder (*p* = 0.035) [48]. In the NEURAPRO cohort, the LA level was inversely correlated with the scores of the Brief Psychiatric Rating Scale (BPRS), especially regarding psychotic symptoms (BPRS psychosis subscale), negative symptoms [Scale for the Assessment of Negative Symptoms (SANS)], manic symptoms [Young Mania Rating Scale (YMRS)], and depressive symptoms [Montgomery Asberg Depression Rating Scale (MADRS)] [49]. In a Polish cohort, converters had a higher consumption of LA than non-converters and healthy controls [50]. 

### 4.3. Sterols in Psychiatry

In our study, the cholestanol/cholesterol ratio improved the prediction of psychotic conversion. The study of the role of cholesterol derivate in membrane metabolism is relatively recent, but several arguments support its clinical relevance. 

The role of membrane cholesterol in psychiatry has been studied mainly in mood disorders. Thus, a post-mortem study found decreased cholesterol levels in the visual cortex (Brodmann area 19) of depressed and bipolar patients. More recently, a study suggested that certain thymoregulators (lithium, valproate, lamotrigine, and quetiapine) have an action on cholesterol metabolism by increasing intracellular cholesterol, which would favour the synthesis of new membranes [51].

The impact of the EPA and DHA on membrane elasticity depends on the membrane cholesterol levels [52]. In addition, cholesterol is an essential component of lipid rafts [53], which notably regulate the activity of the serotonergic and dopaminergic receptors [54,55]. An in vitro study found that clozapine upregulated cholesterol genes and had a huge effect on neuronal cell cholesterol metabolism [56]. Furthermore, the abnormal storage of cholestanol results in cerebrotendinous xanthomatosis, a rare disease exhibiting psychiatric symptoms which were improved by cerebrotendinous xanthomatosis treatment (chenodeoxycholic acid) [57]. The psychiatric diagnoses possible with cerebrotendinous xanthomatosis are varied: anxiety, mood disorder, personality disorder, and psychosis [58]. In a literature review including 194 cases, 17.5% had psychiatric disorders, 35% had cognitive problems at inclusion, and 46.4% had cognitive decline [59]. School difficulties usually start between 5 and 15 years of age, and psychiatric symptoms appear between 15 and 30 years of age [60]. 

Membrane sterols are under-studied in psychosis, and our results indicate that they could be helpful for diagnosis or treatment. A recent study on plasma oxysterols found that the ratios of cholestane-3β,5α,6β-triol, 27-hydroxycholesterol (27-OHC), and cholestanol to total cholesterol increased in drug-free schizophrenic patients compared to controls [61]. Interestingly, a type of psychosis was also linked with cerebral arteriosclerosis; the American Psychiatric Association called it “Chronic Brain Syndrome associated with cerebral arteriosclerosis” [62]. More recently, a review described how oxysterols cause phospholipid packing defects within the membranes of vascular endothelial cells, potentially increasing the cell permeability of low-density lipoprotein cholesterol, which may lead to atherosclerosis [63].

### 4.4. Membrane Lipids for Personalised Medicine

The existence of subgroups could explain the heterogeneity of the results found in UHR patients. The membrane lipids analysis could help distinguish subgroups of patients with specific endophenotypes to allow personalised treatment. As polyunsaturated fatty acids are also linked to oxidative stress and inflammation [1], it could also be interesting to measure these markers. Indeed, depressed patients with a high level of inflammation had a better response to omega-3 supplementation [64]. Interestingly, the levels of alpha-linolenic acid (C18:3n3) at baseline were significantly different between responders and non-responders to omega-3 supplementation (0.21% vs. 0.16%, *p* = 0.001) in the NEUROPRO study [65]. Another study on the NEURAPRO cohort found that complement and coagulation proteins mediate the clinical response to omega-3 PUFA and suggested that an omega-3 increase decreased symptom severity and improved cognition [66].

Because the plasmatic membrane is a complex and dynamic system, the perturbation of one single lipid species can reflect a larger dysregulation in the overall membrane lipid metabolism. This may explain the heterogeneity of the results in the literature and highlight the importance of a broad exploration of membrane lipids. Membrane lipid abnormalities could also be linked with other metabolic pathways that could serve as interesting biomarkers: for instance, lipids could indicate the impact of oxidative stress, inflammation, or C1-metabolism abnormalities [1].

Interestingly, in a study including 327 schizophrenia patients over various episodes (first-episode drug naïve; 2–3 episodes; 4–6 episodes; over 6 episodes) and 159 controls, the membrane fatty acids were only increased in patients within three episodes. Results of fatty acid ratios suggested that the dysregulation of enzymes such as D6 desaturase, D5 desaturase, and elongases for PUFA in patients with multi-episode schizophrenia could account for the differences. An increased ratio of C20:3n6/C18:2n6 was observed in first-episode patients but not in relapsed patients. The C20:4n6/C20:3n6 ratio (D5 desaturase) was decreased after treatment in subgroups, except for the group with over six episodes [67]. More recently, a study on stabilised schizophrenia patients found two subgroups of patients: the DHA group (N = 19) with a lower proportion of membrane DHA as compared to the general population, and the DHA n group (N = 18) with a normal proportion of DHA. DHA patients had more hospitalisations and a lower quality of life than DHA n patients [68].

## 5. Limitations

This study has several limitations. First, the number of subjects is limited, even if the number of converters is higher than in previous reports in the domain. Despite the longitudinal design, we only had data for 23 of the 29 converters at the final time. Larger studies are needed for definitive conclusions, including more converters and non-converters. Second, subjects did not complete a food questionnaire, and we cannot exclude that there was a difference in the intake between the groups. A recent study found that incidence rates of schizophrenia were inversely correlated with arachidonic acid (AA) and omega-6 long-chain polyunsaturated fatty acid consumption [69]. However, the diet may be similar at the inclusion time, as UHR patients have the same level of distress and similar presentation at this time. Finally, the observed differences may not be directly related to the pathophysiology of the conversion to psychosis conversion but rather due to external factors (diet, infections, etc.). However, we believe that this study sheds light on the processes involving the membrane lipids accompanying the onset of the disease.

## 6. Conclusions

Even though the AUC is below 0.9, these results suggest that the analysis of red blood cell membrane lipids might help improve psychotic conversion prediction in UHR patients. Our study is the first to shed light on how the membrane sterols, in association with FA and phospholipids, could improve predicting psychotic conversion. We found that a high level of linoleic acid (omega-6) was associated with an increased risk of psychotic transition. Because omega-6 and omega-3 had antagonist properties, this provides a rationale to explain why omega-3 supplementation could help prevent psychosis, at least in a subgroup of patients. Future omega-3 supplementation studies in UHR or FEP patients should include initial lipid profiles to determine the need for supplementation to advance personalised medicine.

## Figures and Tables

**Table 1:** Clinical characteristics of the population at the time of inclusion. BMI: Body mass index; CRP: C-reactive protein ultra-sensible; GLY: Glycaemia; HDL: High-density lipoprotein cholesterol; TRIG: Triglycerides; SOFAS: Social and Occupational Functioning Assessment Scale; PANSS: Positive And Negative Symptoms Scale; SAPS: Scale for the Assessment of Positive Symptoms; SANS: Scale for the Assessment of Negative Symptoms; MADRS: Montgomery and Asberg Depression Rating Scale; CPZ EQ: Chlorpromazine equivalent; THC 30 days: Cannabis use in the last month.

	Converters		Non-Converters		*p*-Value
	N	Mean (SD)	N	Mean (SD)	
Age (year)	29	20.21 (2.43)	32	21.5 (3.46)	0.10
BMI	29	21.98 (3.49)	32	21.9 (3.06)	0.92
CRP	22	2.3 (3.85)	28	2.21 (2.44)	0.92
GLY	24	4.57 (0.93)	30	4.62 (0.92)	0.83
HDL	25	1.21 (0.46)	27	1.32 (0.37)	0.33
TRIG	25	1.02 (0.59)	29	0.99 (0.53)	0.88
SOFAS	28	48.04 (10.13)	32	46.75 (9.25)	0.61
PANSSTOT	28	67.86 (25.3)	32	71.72 (17)	0.49
SANS	24	23.83 (18.01)	32	22.63 (16)	0.79
SAPS	24	16.75 (14.54)	32	13.28 (9.18)	0.28
MADRS	24	20.5 (7.35)	32	21.19 (9.31)	0.77
CPZ EQ	24	23.88 (43.91)	32	17.77 (46.8)	0.62
	N	Percent	N	Percent	
Men (%)	17	58.60%	18	56.30%	0.91
Cannabis use last month					0.55 *
0	13	45%	18	62%	
1–2	2	7%	1	3%	
3–9	4	14%	1	3%	
>10	3	10%	5	17%	
NA	7	24%	7	15%	

*: using the Fisher test.

**Table 2:** Correlation between membrane lipids and plasmatic lipids. PUFA: Polyunsaturated fatty acids; PC: Phosphatidylcholine; PE: Phosphatidylethanolamine; PS: Phosphatidylserine; SM: Sphingomyelin.

Membrane Lipids (%)	Body Mass Index Rho (Spearman)	*p*	Plasmatic Cholesterol Rho (Spearman)	*p*	Triglycerides Rho (Spearman)	*p*
Omega-3	−0.08	0.53	0.06	0.66	−0.40	0.003
Omega-6	−0.07	0.57	0.15	0.30	−0.25	0.07
Omega-9	0.12	0.38	−0.04	0.76	0.33	0.02
Total PUFA	−0.07	0.62	0.09	0.51	−0.37	0.005
Cholestanol	−0.01	0.95	−0.25	0.08	0.16	0.28
Cholesterol	0.08	0.57	−0.12	0.42	0.10	0.47
PC	0.14	0.27	−0.03	0.84	0.21	0.13
PE	−0.24	0.06	0.12	0.38	−0.02	0.91
PS	−0.04	0.75	0.06	0.69	0.04	0.77
SM	−0.07	0.57	−0.21	0.12	−0.12	0.40

**Table 3:** Linoleic acid (LA) levels regarding the transition status. *p* = 0.02 (Fischer test).

	Low LA Level	High LA Level	Total
Converters	6 (26%)	23 (61%)	29
Non-converters	17 (74%)	15 (39%)	32
Total	23	38	61

**Table 4:** Comparison of mean fatty acid levels regarding the linoleic acid (LA) level cluster using the Wilcoxon test and Benjamin Hochberg adjustment.

	Low LA Level	Low LA Level	*p*-Value	*p*-Value Adjusted
**C14_0**	0.275217	0.331053	0.2	0.35556
**C16_0**	22.26739	22.01053	0.7	0.74667
**C16_1**	0.733043	0.740263	0.6	0.74667
**C18_0**	17.962174	16.466842	0.001	0.00320
**C18_1n9**	16.847826	17.370526	0.4	0.58182
**C18_1n7**	1.175217	1.160789	0.7	0.74667
**C18_3n6**	0.086957	0.084474	0.7	0.74667
**C18_3n3**	0.123043	0.179211	0.0005	0.00267
**C20_3n9**	0.295652	0.283158	0.3	0.48000
**C20_3n6**	1.616957	1.625263	0.8	0.80000
**C20_4n6**	15.975652	13.988421	0.001	0.00320
**C20_5n3**	0.678696	0.603158	0.07	0.14000
**C22_4n6**	2.83	2.194737	0.0003	0.00267
**C22_5n6**	0.516087	0.427368	0.05	0.11429
**C22_5n3**	2.184783	1.737632	0.0004	0.00267
**C22_6n3**	4.635652	3.984737	0.05	0.11429

**Table 5:** Comparison of mean lipid percentages between the inclusion time and the final time before adjustment using Wilcoxon test. LPC: Lyso Phosphatidyl Choline; PS: Phosphatidyl Serine.

	Inclusion	Final Time	*p*
C16_0	22.107377	22.95463	0.002
C18_3n6	0.08541	0.134074	<0.001
LPC(18:3)	0.006299	0.006612	0.050
PS 32:0	0.33473	0.33627	0.045
PS 36:0	0.002418	0.007212	<0.001

**Table 6:** Comparison of longitudinal changes in mean lipid percentages between converters and non-converters before adjustment using the Wilcoxon test. LPC: Lyso Phosphatidyl Choline; PC: Phosphatidyl Choline; Cer: Ceramide; LactoCer: Lacto Ceramides.

	Converters	Non-Converters	*p*
C22_5n6	0.003209	−0.006645	0.005
LPC(18:3)	0.000216	−0.000042	0.04
PC O34:1	0.00128	−0.002841	0.03
PC 38-3	0.006624	−0.000174	0.04
Cer d18:1/22:2—H2O	0.008777	−0.000666	0.02
Cer d18:1/16:0—H2O	0.018843	−0.021831	0.05
LactoCer d18:1/12:0—H2O	0.00433	−0.001459	0.03

## Data Availability

Restrictions apply to the availability of these data because of French legislation. Please contact marie-odile.krebs@inserm.fr for more informations.

## Supplementary Materials

The following supporting information can be downloaded at: https://www.mdpi.com/article/10.3390/nu15092215/s1, Supplementary Figure S1: Heatmap for fatty acids showing only group averages at inclusion regarding the status; Supplementary Figure S2: Heatmap for phospholipids showing only group averages at inclusion regarding the status; Supplementary Figure S3: Heatmap for fatty acids showing only group averages at inclusion regarding the cluster of Linoleic acid (LA) level; Supplementary Figure S4: Heatmap for membrane lipids showing only group averages for all subjects regarding time. Class 0: inclusion. Class 1: final time; Supplementary Figure S5: Heatmap for membrane lipids showing only group averages for converters regarding time. Class 0: inclusion. Class 1: final time; Supplementary Figure S6: Heatmap for membrane lipids showing only group averages for non converters regarding time. Class 0: inclusion. Class 1: final time.
