# Supplementary material for: Membrane Lipids in Ultra-High-Risk Patients: Potential Predictive Biomarkers of Conversion to Psychosis
Source: Nutrients. 2023 May 7;15(9):2215. doi: 10.3390/nu15092215 (PMC10181001; doi:10.3390/nu15092215)
Supplement: Supplementary file 1 [file nutrients-15-02215-s001.zip › nutrients-2354509-supplementary.pdf]

Supplementary Figure S1 : Heatmap for fatty acids showing only group averages at inclusion regarding the status

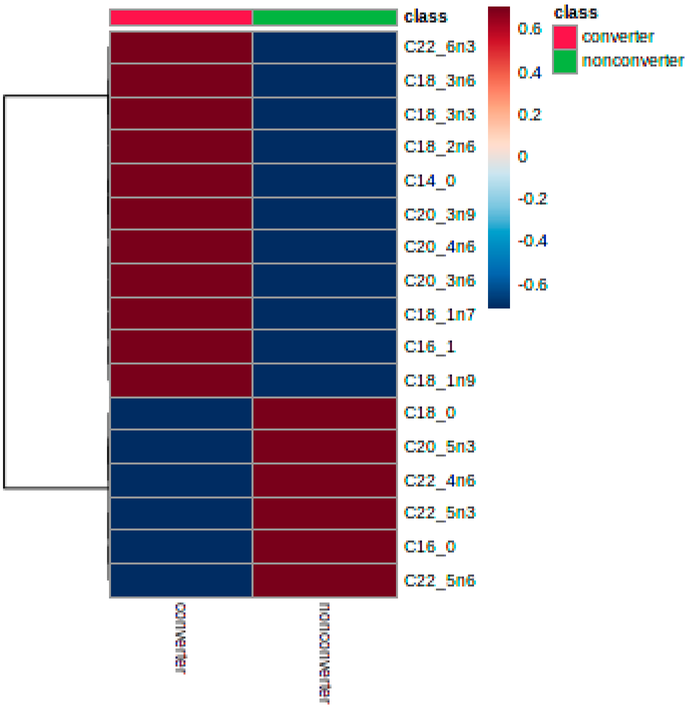

Supplementary Figure S2 : Heatmap for phospholipids showing only group averages at inclusion regarding the status

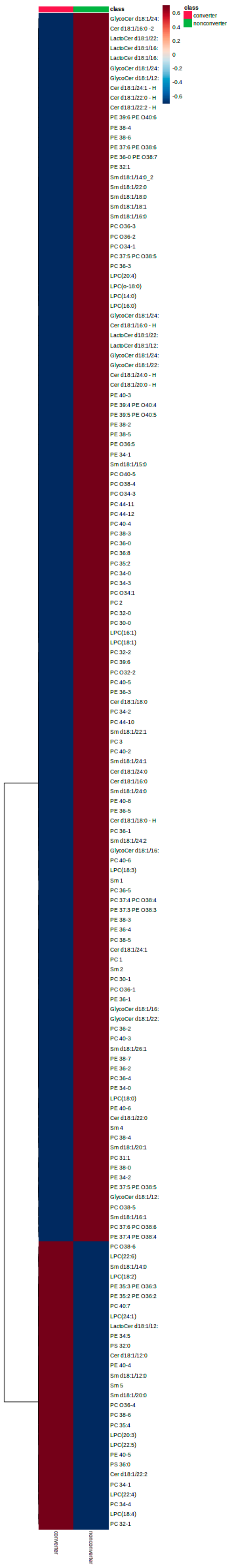
class
GlycoCer d18:1/24:
Cer d18:1/16:0 -2
LactoCer d18:1/22:
LactoCer d18:1/16:
LactoCer d18:1/16:
GlycoCer d18:1/24:
GlycoCer d18:1/12:
Cer d18:1/24:1 - H
Cer d18:1/22:0 - H
Cer d18:1/22:2 - H
PE 39:6 PE O40:6
PE 38-4
PE 38-6
PE 37:6 PE O38:6
PE 36-0 PE O38:7
PE 32:1
Sm d18:1/14:0_2
Sm d18:1/22:0
Sm d18:1/18:0
Sm d18:1/18:1
Sm d18:1/16:0
PC O36-3
PC O36-2
PC O34-1
PC 37:5 PC O38:5
PC 36-3
LPC(20:4)
LPC(o-18:0)
LPC(14:0)
LPC(16:0)
GlycoCer d18:1/24:
Cer d18:1/16:0 - H
LactoCer d18:1/22:
LactoCer d18:1/12:
GlycoCer d18:1/24:
GlycoCer d18:1/22:
Cer d18:1/24:0 - H
Cer d18:1/20:0 - H
PE 40-3
PE 39:4 PE O40:4
PE 39:5 PE O40:5
PE 38-2
PE 38-5
PE O36:5
PE 34-1
Sm d18:1/15:0
PC O40-5
PC O38-4
PC O34-3
PC 44-11
PC 44-12
PC 40-4
PC 38-3
PC 36-0
PC 36:8
PC 35:2
PC 34-0
PC 34-3
PC O34:1
PC 2
PC 32-0
PC 30-0
LPC(16:1)
LPC(18:1)
PC 32-2
PC 39:6
PC O32-2
PC 40-5
PE 36-3
Cer d18:1/18:0
PC 34-2
PC 44-10
Sm d18:1/22:1
PC 3
PC 40-2
Sm d18:1/24:1
Cer d18:1/24:0
Cer d18:1/16:0
Sm d18:1/24:0
PE 40-8
PE 36-5
Cer d18:1/18:0 - H
PC 36-1
Sm d18:1/24:2
GlycoCer d18:1/16:
PC 40-6
LPC(18:3)
Sm 1
PC 36-5
PC 37:4 PC O38:4
PE 37:3 PE O38:3
PE 38-3
PE 36-4
PC 38-5
Cer d18:1/24:1
PC 1
Sm 2
PC 30-1
PC O36-1
PE 36-1
GlycoCer d18:1/16:
GlycoCer d18:1/22:
PC 36-2
PC 40-3
Sm d18:1/26:1
PE 38-7
PE 36-2
PC 36-4
PE 34-0
LPC(18:0)
PE 40-6
Cer d18:1/22:0
Sm 4
PC 38-4
Sm d18:1/20:1
PC 31:1
PE 38-0
PE 34-2
PE 37:5 PE O38:5
GlycoCer d18:1/12:
PC O38-5
Sm d18:1/16:1
PC 37:6 PC O38:6
PE 37:4 PE O38:4
PC O38-6
LPC(22:6)
Sm d18:1/14:0
LPC(18:2)
PE 35:3 PE O36:3
PE 35:2 PE O36:2
PC 40:7
LPC(24:1)
LactoCer d18:1/12:
PE 34:5
PS 32:0
Cer d18:1/12:0
PE 40-4
Sm d18:1/12:0
Sm 5
Sm d18:1/20:0
PC O36-4
PC 38-6
PC 35:4
LPC(20:3)
LPC(22:5)
PE 40-5
PS 36:0
Cer d18:1/22:2
PC 34-1
LPC(22:4)
PC 34-4
LPC(18:4)
PC 32-1
converter
nonconverter
0.6
0.4
0.2
0
-0.2
-0.4
-0.6
class
converter
nonconverter

Supplementary Figure S3 : Heatmap for fatty acids showing only group averages at inclusion regarding the cluster of Linoleic acid (LA) level

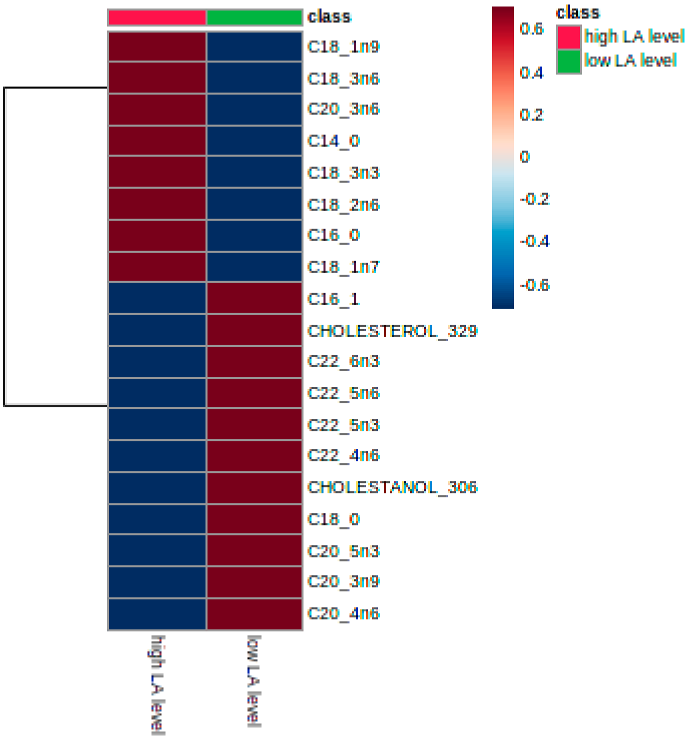

Supplementary Figure S4 : Heatmap for membrane lipids showing only group averages for all subjects regarding time. Class 0: inclusion. Class 1: final time

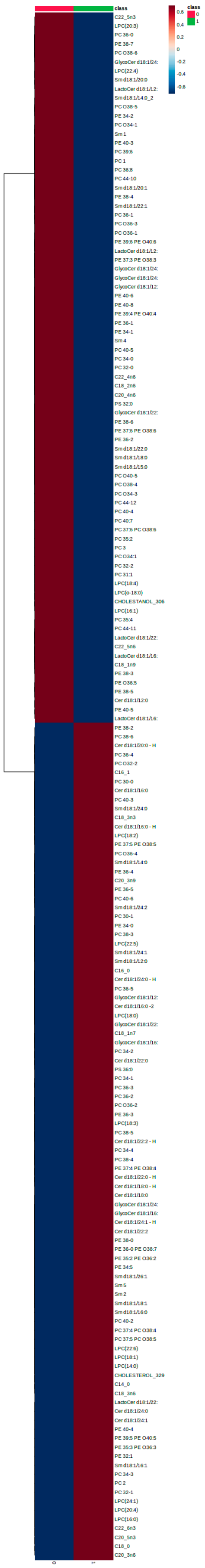
class
C22_5n3
LPC(20:3)
PC 36-0
PE 38-7
PC O38-6
GlycoCer d18:1/24:
LPC(22:4)
Sm d18:1/20:0
LactoCer d18:1/12:
Sm d18:1/14:0_2
PC O38-5
PE 34-2
PC O34-1
Sm 1
PE 40-3
PC 39:6
PC 1
PC 36:8
PC 44-10
Sm d18:1/20:1
PE 38-4
Sm d18:1/22:1
PC 36-1
PC O36-3
PC O36-1
PE 39:6 PE O40:6
LactoCer d18:1/12:
PE 37:3 PE O38:3
GlycoCer d18:1/24:
GlycoCer d18:1/24:
GlycoCer d18:1/12:
PE 40-6
PE 40-8
PE 39:4 PE O40:4
PE 36-1
PE 34-1
Sm 4
PC 40-5
PC 34-0
PC 32-0
C22_4n6
C18_2n6
C20_4n6
PS 32:0
GlycoCer d18:1/22:
PE 38-6
PE 37:6 PE O38:6
PE 36-2
Sm d18:1/22:0
Sm d18:1/18:0
Sm d18:1/15:0
PC O40-5
PC O38-4
PC O34-3
PC 44-12
PC 40-4
PC 40:7
PC 37:6 PC O38:6
PC 35:2
PC 3
PC O34:1
PC 32-2
PC 31:1
LPC(18:4)
LPC(o-18:0)
CHOLESTANOL_306
LPC(16:1)
PC 35:4
PC 44-11
LactoCer d18:1/22:
C22_5n6
LactoCer d18:1/16:
C18_1n9
PE 38-3
PE O36:5
PE 38-5
Cer d18:1/12:0
PE 40-5
LactoCer d18:1/16:
PE 38-2
PC 38-6
Cer d18:1/20:0 - H
PC 36-4
PC O32-2
C16_1
PC 30-0
Cer d18:1/16:0
PC 40-3
Sm d18:1/24:0
C18_3n3
Cer d18:1/16:0 - H
LPC(18:2)
PE 37:5 PE O38:5
PC O36-4
Sm d18:1/14:0
PE 36-4
C20_3n9
PE 36-5
PC 40-6
Sm d18:1/24:2
PC 30-1
PE 34-0
PC 38-3
LPC(22:5)
Sm d18:1/24:1
Sm d18:1/12:0
C16_0
Cer d18:1/24:0 - H
PC 36-5
GlycoCer d18:1/12:
Cer d18:1/16:0 -2
LPC(18:0)
GlycoCer d18:1/22:
C18_1n7
GlycoCer d18:1/16:
PC 34-2
Cer d18:1/22:0
PS 36:0
PC 34-1
PC 36-3
PC 36-2
PC O36-2
PE 36-3
LPC(18:3)
PC 38-5
Cer d18:1/22:2 - H
PC 34-4
PC 38-4
PE 37:4 PE O38:4
Cer d18:1/22:0 - H
Cer d18:1/18:0 - H
Cer d18:1/18:0
GlycoCer d18:1/24:
GlycoCer d18:1/16:
Cer d18:1/24:1 - H
Cer d18:1/22:2
PE 38-0
PE 36-0 PE O38:7
PE 35:2 PE O36:2
PE 34:5
Sm d18:1/26:1
Sm 5
Sm 2
Sm d18:1/18:1
Sm d18:1/16:0
PC 40-2
PC 37:4 PC O38:4
PC 37:5 PC O38:5
LPC(22:6)
LPC(18:1)
LPC(14:0)
CHOLESTEROL_329
C14_0
C18_3n6
LactoCer d18:1/22:
Cer d18:1/24:0
Cer d18:1/24:1
PE 40-4
PE 39:5 PE O40:5
PE 35:3 PE O36:3
PE 32:1
Sm d18:1/16:1
PC 34-3
PC 2
PC 32-1
LPC(24:1)
LPC(20:4)
LPC(16:0)
C22_6n3
C20_5n3
C18_0
C20_3n6
0
1
0.6
0.4
0.2
0
-0.2
-0.4
-0.6
class
0
1

Supplementary Figure S5 : Heatmap for membrane lipids showing only group averages for converters regarding time. Class 0: inclusion. Class 1: final time

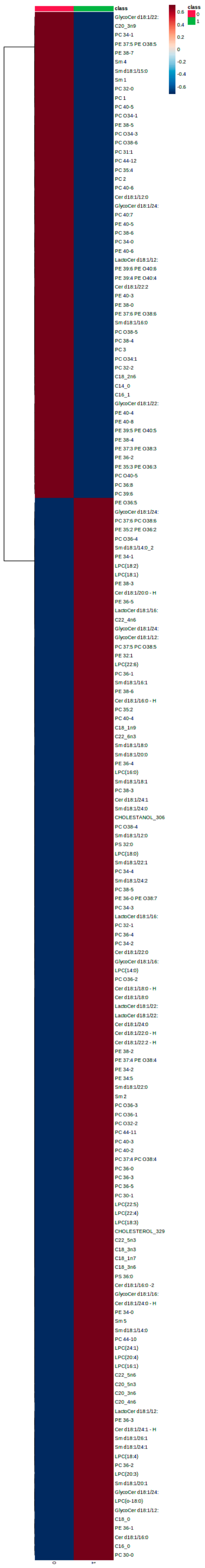

class
GlycoCer d18:1/22:
C20_3n9
PC 34-1
PE 37:5 PE O38:5
PE 38-7
Sm 4
Sm d18:1/15:0
Sm 1
PC 32-0
PC 1
PC 40-5
PC O34-1
PE 38-5
PC O34-3
PC O38-6
PC 31:1
PC 44-12
PC 35:4
PC 2
PC 40-6
Cer d18:1/12:0
GlycoCer d18:1/24:
PC 40:7
PE 40-5
PC 38-6
PC 34-0
PE 40-6
LactoCer d18:1/12:
PE 39:6 PE O40:6
PE 39:4 PE O40:4
Cer d18:1/22:2
PE 40-3
PE 38-0
PE 37:6 PE O38:6
Sm d18:1/16:0
PC O38-5
PC 38-4
PC 3
PC O34:1
PC 32-2
C18_2n6
C14_0
C16_1
GlycoCer d18:1/22:
PE 40-4
PE 40-8
PE 39:5 PE O40:5
PE 38-4
PE 37:3 PE O38:3
PE 36-2
PE 35:3 PE O36:3
PC O40-5
PC 36:8
PC 39:6
PE O36:5
GlycoCer d18:1/24:
PC 37:6 PC O38:6
PE 35:2 PE O36:2
PC O36-4
Sm d18:1/14:0_2
PE 34-1
LPC(18:2)
LPC(18:1)
PE 38-3
Cer d18:1/20:0 - H
PE 36-5
LactoCer d18:1/16:
C22_4n6
GlycoCer d18:1/24:
GlycoCer d18:1/12:
PC 37:5 PC O38:5
PE 32:1
LPC(22:6)
PC 36-1
Sm d18:1/16:1
PE 38-6
Cer d18:1/16:0 - H
PC 35:2
PC 40-4
C18_1n9
C22_6n3
Sm d18:1/18:0
Sm d18:1/20:0
PE 36-4
LPC(16:0)
Sm d18:1/18:1
PC 38-3
Cer d18:1/24:1
Sm d18:1/24:0
CHOLESTANOL_306
PC O38-4
Sm d18:1/12:0
PS 32:0
LPC(18:0)
Sm d18:1/22:1
PC 34-4
Sm d18:1/24:2
PC 38-5
PE 36-0 PE O38:7
PC 34-3
LactoCer d18:1/16:
PC 32-1
PC 36-4
PC 34-2
Cer d18:1/22:0
GlycoCer d18:1/16:
LPC(14:0)
PC O36-2
Cer d18:1/18:0 - H
Cer d18:1/18:0
LactoCer d18:1/22:
LactoCer d18:1/22:
Cer d18:1/24:0
Cer d18:1/22:0 - H
Cer d18:1/22:2 - H
PE 38-2
PE 37:4 PE O38:4
PE 34-2
PE 34:5
Sm d18:1/22:0
Sm 2
PC O36-3
PC O36-1
PC O32-2
PC 44-11
PC 40-3
PC 40-2
PC 37:4 PC O38:4
PC 36-0
PC 36-3
PC 36-5
PC 30-1
LPC(22:5)
LPC(22:4)
LPC(18:3)
CHOLESTEROL_329
C22_5n3
C18_3n3
C18_1n7
C18_3n6
PS 36:0
Cer d18:1/16:0 -2
GlycoCer d18:1/16:
Cer d18:1/24:0 - H
PE 34-0
Sm 5
Sm d18:1/14:0
PC 44-10
LPC(24:1)
LPC(20:4)
LPC(16:1)
C22_5n6
C20_5n3
C20_3n6
C20_4n6
LactoCer d18:1/12:
PE 36-3
Cer d18:1/24:1 - H
Sm d18:1/26:1
Sm d18:1/24:1
LPC(18:4)
PC 36-2
LPC(20:3)
Sm d18:1/20:1
GlycoCer d18:1/24:
LPC(o-18:0)
GlycoCer d18:1/12:
C18_0
PE 36-1
Cer d18:1/16:0
C16_0
PC 30-0
0.6
0.4
0.2
0
-0.2
-0.4
-0.6
class
0
1
0
1

Supplementary Figure S6 : Heatmap for membrane lipids showing only group averages for non converters regarding time. Class 0: inclusion. Class 1: final time

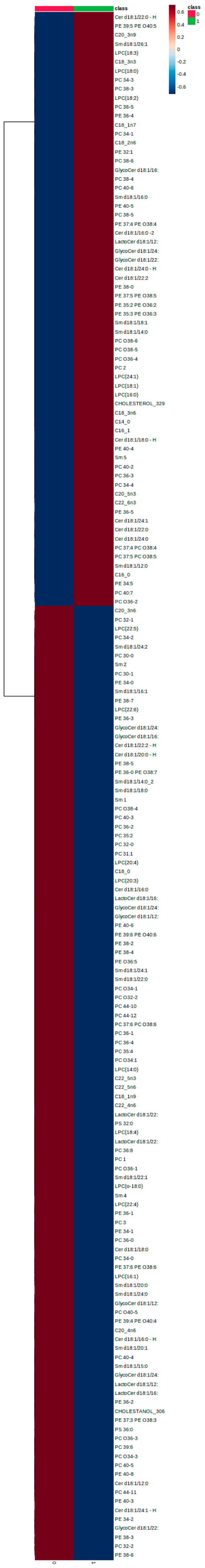
class
0.6
0.4
0.2
0
-0.2
-0.4
-0.6
class
0
1
Cer d18:1/22:0 - H
PE 39:5 PE O40:5
C20_3n9
Sm d18:1/26:1
LPC(18:3)
C18_3n3
LPC(18:0)
PC 34-3
PC 38-3
LPC(18:2)
PC 36-5
PE 36-4
C18_1n7
PC 34-1
C18_2n6
PE 32:1
PC 38-6
GlycoCer d18:1/16:
PC 38-4
PC 40-6
Sm d18:1/16:0
PE 40-5
PC 38-5
PE 37:4 PE O38:4
Cer d18:1/16:0 -2
LactoCer d18:1/12:
GlycoCer d18:1/24:
GlycoCer d18:1/22:
Cer d18:1/24:0 - H
Cer d18:1/22:2
PE 38-0
PE 37:5 PE O38:5
PE 35:2 PE O36:2
PE 35:3 PE O36:3
Sm d18:1/18:1
Sm d18:1/14:0
PC O38-6
PC O38-5
PC O36-4
PC 2
LPC(24:1)
LPC(18:1)
LPC(16:0)
CHOLESTEROL_329
C18_3n6
C14_0
C16_1
Cer d18:1/18:0 - H
PE 40-4
Sm 5
PC 40-2
PC 36-3
PC 34-4
C20_5n3
C22_6n3
PE 36-5
Cer d18:1/24:1
Cer d18:1/22:0
Cer d18:1/24:0
PC 37:4 PC O38:4
PC 37:5 PC O38:5
Sm d18:1/12:0
C16_0
PE 34:5
PC 40:7
PC O36-2
C20_3n6
PC 32-1
LPC(22:5)
PC 34-2
Sm d18:1/24:2
PC 30-0
Sm 2
PC 30-1
PE 34-0
Sm d18:1/16:1
PE 38-7
LPC(22:6)
PE 36-3
GlycoCer d18:1/24:
GlycoCer d18:1/16:
Cer d18:1/22:2 - H
Cer d18:1/20:0 - H
PE 38-5
PE 36-0 PE O38:7
Sm d18:1/14:0_2
Sm d18:1/18:0
Sm 1
PC O38-4
PC 40-3
PC 36-2
PC 35:2
PC 32-0
PC 31:1
LPC(20:4)
C18_0
LPC(20:3)
Cer d18:1/16:0
LactoCer d18:1/16:
GlycoCer d18:1/24:
GlycoCer d18:1/12:
PE 40-6
PE 39:6 PE O40:6
PE 38-2
PE 38-4
PE O36:5
Sm d18:1/24:1
Sm d18:1/22:0
PC O34-1
PC O32-2
PC 44-10
PC 44-12
PC 37:6 PC O38:6
PC 36-1
PC 36-4
PC 35:4
PC O34:1
LPC(14:0)
C22_5n3
C22_5n6
C18_1n9
C22_4n6
LactoCer d18:1/22:
PS 32:0
LPC(18:4)
LactoCer d18:1/22:
PC 36:8
PC 1
PC O36-1
Sm d18:1/22:1
LPC(o-18:0)
Sm 4
LPC(22:4)
PE 36-1
PC 3
PE 34-1
PC 36-0
Cer d18:1/18:0
PC 34-0
PE 37:6 PE O38:6
LPC(16:1)
Sm d18:1/20:0
Sm d18:1/24:0
GlycoCer d18:1/12:
PC O40-5
PE 39:4 PE O40:4
C20_4n6
Cer d18:1/16:0 - H
Sm d18:1/20:1
PC 40-4
Sm d18:1/15:0
GlycoCer d18:1/24:
LactoCer d18:1/12:
LactoCer d18:1/16:
PE 36-2
CHOLESTANOL_306
PE 37:3 PE O38:3
PS 36:0
PC O36-3
PC 39:6
PC O34-3
PC 40-5
PE 40-8
Cer d18:1/12:0
PC 44-11
PE 40-3
Cer d18:1/24:1 - H
PE 34-2
GlycoCer d18:1/22:
PE 38-3
PC 32-2
PE 38-6
0
1
